# Primary Dural Diffuse Large B-Cell Lymphoma: A Report of a Rare Case and Review of the Literature

**DOI:** 10.7759/cureus.102290

**Published:** 2026-01-26

**Authors:** Adetola G Mowo-wale, Ozo Akah, Shaileshkumar Jagdishbhai Prajapati, Parita Shah, Kaushalendra Mani Tripathi, Neeraj Kancherla, Rohan Raj

**Affiliations:** 1 Internal Medicine, Obafemi Awolowo College of Health Sciences, Sagamu, NGA; 2 Internal Medicine, Carnegie Mellon University, Houston, USA; 3 Internal Medicine, Penza State University, Penza, RUS; 4 Internal Medicine, Surat Municipal Institute of Medical Education and Research, Surat, IND; 5 Internal Medicine, White River Health Medical Center, Batesville, USA; 6 Internal Medicine, Philadelphia College of Osteopathic Medicine, Philadelphia, USA; 7 Internal Medicine, Nalanda Medical College and Hospital, Patna, IND

**Keywords:** case report, cns lymphoma, diffuse large b-cell lymphoma, meningioma mimic, primary dural lymphoma, r-chop

## Abstract

Primary dural lymphoma (PDL) is a rare subtype of primary central nervous system lymphoma (PCNSL), accounting for fewer than 1% of cases. It originates from the dura mater with no evidence of parenchymal or systemic involvement and is usually a low-grade marginal zone B-cell lymphoma (MZL). High-grade variants, including diffuse large B-cell lymphoma (DLBCL), are extremely uncommon and clinically more aggressive. We describe the case of a 65-year-old immunocompetent man who presented with progressive headaches and right-sided weakness. Brain MRI revealed a right frontoparietal dural-based enhancing lesion with a dural tail, closely mimicking meningioma on imaging. Subtotal resection was performed, and histopathology showed large atypical B cells positive for CD20, CD79a, CD10, and BCL6, with a Ki-67 index greater than 60%, confirming a diagnosis of primary dural DLBCL. The patient received six cycles of rituximab-based chemotherapy (R-CHOP: rituximab, cyclophosphamide, doxorubicin, vincristine, prednisone), followed by cranial radiotherapy, and achieved complete remission after 12 months.

PDL often imitates meningioma on imaging, which may lead to diagnostic delays. While MZL subtypes typically behave indolently, DLBCL variants require aggressive multimodal therapy. This report highlights the importance of histopathologic confirmation in all dural-based lesions and demonstrates that early multidisciplinary management, including surgery, rituximab-based chemotherapy, and radiotherapy, can result in sustained remission even in highly aggressive forms.

## Introduction

Primary dural lymphoma (PDL) is an exceedingly rare neoplasm arising from the dura mater without initial involvement of the brain parenchyma or systemic disease. It represents less than 1% of primary central nervous system lymphomas (PCNSL) and approximately 0.1% of all non-Hodgkin lymphomas [1,2]. The majority of PDLs are low-grade marginal zone B-cell lymphomas (MZL) that follow an indolent course and respond well to localized therapy, such as surgical excision or focal irradiation [3]. In contrast, high-grade subtypes, such as diffuse large B-cell lymphoma (DLBCL), are uncommon and carry a poorer prognosis, necessitating systemic chemoimmunotherapy [4]. Clinically and radiographically, PDLs often resemble meningiomas because of their dural attachment, homogeneous enhancement, and dural tail on MRI [5]. This resemblance often leads to misdiagnosis and delayed treatment. The diagnosis requires histopathologic and immunohistochemical confirmation following surgical biopsy or resection. We present a case of primary dural DLBCL in an immunocompetent 65-year-old man, initially presumed to harbor a meningioma. We also provide a focused review of contemporary literature emphasizing diagnostic differentiation and optimal therapeutic strategies.

Written informed consent was obtained from the patient for publication of this case report and the accompanying images.

## Case presentation

A 65-year-old right-handed male presented with a two-month history of worsening holocranial headaches and intermittent vomiting. He also reported mild weakness in the right upper limb but denied seizures, visual disturbances, or cognitive decline. His medical history was unremarkable, with no evidence of prior malignancy, immunosuppression, or systemic “B” symptoms. On examination, the patient was alert and oriented with a Glasgow Coma Scale (GCS) score of 15. Neurological assessment revealed a right pronator drift (Medical Research Council grade 4/5 strength) and mild right lower limb hyperreflexia. There was no cranial nerve deficit or papilledema. Systemic examination was normal. Routine blood tests were within reference limits except for a mildly elevated lactate dehydrogenase (LDH) level of 250 U/L (normal: <200 U/L).

**Figure 1:**
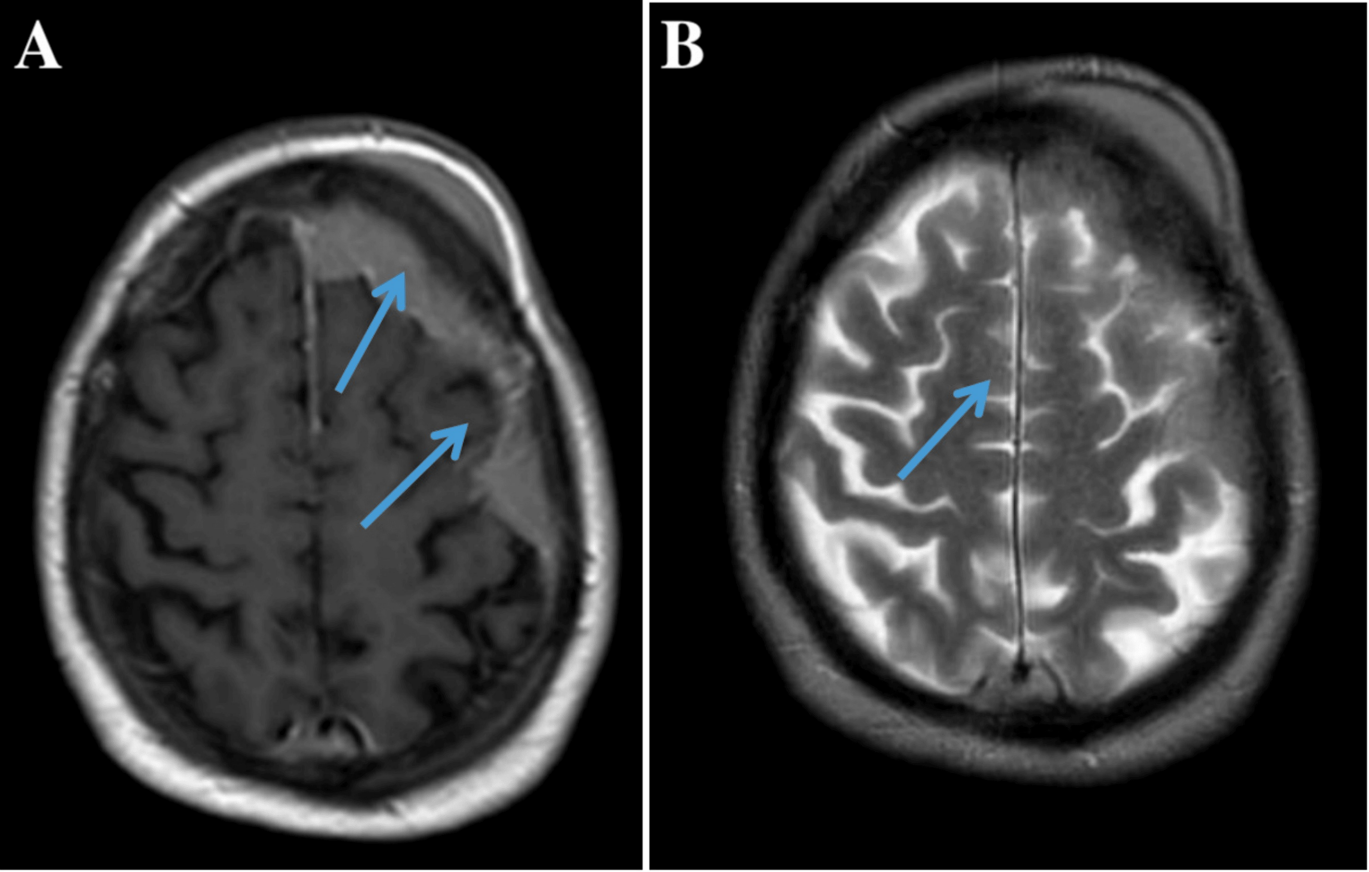
Axial MRI of the right frontoparietal convexity demonstrating an extra-axial dural-based enhancing mass with a dural tail sign (A) T1-weighted post-contrast image showing the homogeneously enhancing mass (arrow) with adjacent dural tail (arrowhead) along the convexity, indicating meningeal involvement. (B) T2-weighted image providing better visualization of the mass's relationship to surrounding cerebrospinal fluid and cortical gyri (arrow), with preserved sulcal spaces suggesting no significant mass effect MRI: magnetic resonance imaging

Contrast-enhanced brain MRI demonstrated a well-circumscribed, extra-axial dural-based lesion measuring 4.2 × 3.8 × 2.5 cm in the right frontoparietal region. The mass was isointense on T1-weighted images and hyperintense on T2/FLAIR sequences, with homogenous post-contrast enhancement and a conspicuous dural tail (Figure 1). Moderate perilesional edema caused a 5-mm midline shift but no brain parenchymal invasion. Diffusion-weighted imaging showed restricted diffusion (ADC = 0.7 × 10⁻³ mm²/s), consistent with hypercellularity. CT of the brain revealed a hyperdense lesion without bone destruction or hyperostosis. Whole-body PET-CT and bone marrow biopsy excluded systemic involvement.

The patient underwent a right frontoparietal craniotomy with subtotal tumor excision (Simpson grade II). Intraoperatively, the lesion appeared firm, grayish-white, and dural-based, easily separable from the underlying cortex. A small dural remnant adherent to the falx cerebri was left to preserve venous structures. Estimated blood loss was 150 mL, and recovery was uneventful.

**Figure 2:**
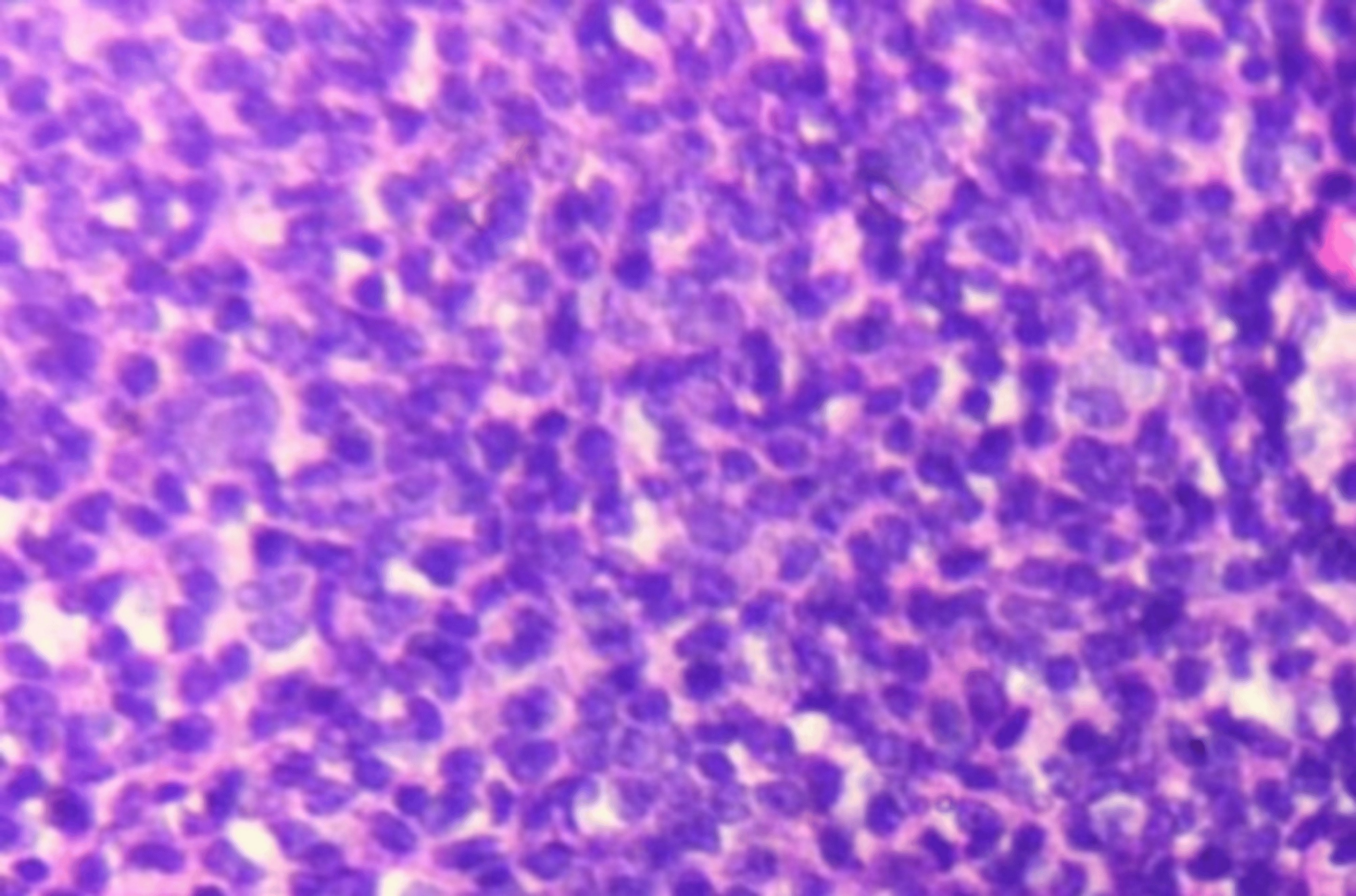
Photomicrograph (H&E ×400) showing diffuse sheets of large atypical lymphoid cells with hyperchromatic nuclei and frequent mitoses H&E: hematoxylin and eosin

Microscopic examination of the resected specimen showed diffuse infiltration by large atypical lymphoid cells with vesicular chromatin, prominent nucleoli, and frequent mitotic figures (Figure 2). Immunohistochemistry was positive for CD20, CD79a, CD10, and BCL6 and negative for CD3, CD5, CD30, EMA, and MUM1. The Ki-67 proliferation index was approximately 65%. Fluorescence in situ hybridization (FISH) excluded MYC rearrangement. These findings confirmed the diagnosis of primary dural diffuse large B-cell lymphoma (non-germinal center subtype).

The patient recovered without neurological deficits. Based on a multidisciplinary tumor board recommendation, he received six cycles of R-CHOP chemotherapy (rituximab, cyclophosphamide, doxorubicin, vincristine, prednisone) over six months, followed by cranial radiotherapy (36 Gy in 20 fractions). He tolerated therapy well, achieving complete radiological remission at 12 months. Surveillance imaging every six months has shown no evidence of disease to date (Karnofsky Performance Status = 90).

## Discussion

PDL represents a rare and distinct clinicopathologic entity within the spectrum of PCNSL. Fewer than 200 cases have been reported worldwide to date [1,6]. It predominantly affects middle-aged and older adults, with a female predominance (3:1 ratio), and most frequently involves the cerebral convexities, falx cerebri, or tentorium cerebelli [2,3]. The dura mater lacks lymphoid tissue under normal conditions, which suggests that chronic inflammation or ectopic lymphoid aggregation may initiate lymphomagenesis [7]. Most PDLs are of MZL histology, characterized by low proliferative activity and favorable outcomes. In contrast, DLBCL variants, such as in this case, are aggressive, displaying a high Ki-67 index (>50%) and necessitating systemic therapy.

PDL closely mimics meningioma radiographically, exhibiting an extra-axial location, homogeneous enhancement, and a dural tail. Distinguishing features that may suggest lymphoma include marked diffusion restriction on DWI, absence of calcification or bone reaction on CT, and rapid clinical progression. Advanced MRI techniques, including perfusion and spectroscopy, may aid in preoperative differentiation by demonstrating low perfusion and elevated choline peaks in lymphoma [8]. Ultimately, tissue diagnosis is essential. There is no consensus on the optimal management of PDL due to its rarity. For low-grade MZL, gross total excision followed by localized radiotherapy often suffices [9]. For DLBCL, however, most authors advocate for multimodal therapy combining surgery, rituximab-based chemotherapy, and focal radiotherapy [4,10]. Intrathecal chemotherapy is generally unnecessary when cerebrospinal fluid is negative for malignant cells.

Recent studies have reported encouraging outcomes with combined therapy. Susanibar-Adaniya and Barta observed an overall survival rate of 85% with R-CHOP plus radiotherapy [11]. Our patient’s durable remission supports this multimodal approach. Prognosis depends on histologic subtype, extent of resection, and adjuvant therapy. MZL variants achieve greater than 90% five-year survival, while DLBCL types show 60-80% with appropriate systemic treatment [2,4]. Unlike parenchymal PCNSL, which carries a guarded outlook, PDL typically remains localized and responds favorably to therapy. Important differential diagnoses include meningioma, metastasis, infectious or inflammatory pachymeningitis, and dural sarcoma. In this case, PET-CT and bone marrow evaluation excluded the presence of systemic lymphoma. Immunohistochemistry confirmed B-cell lineage and ruled out epithelial or T-cell neoplasms.

The principal strength of this case report is the comprehensive clinicoradiologic-pathologic characterization of a rare aggressive variant of PDL, specifically diffuse large B-cell lymphoma, within a cohort of fewer than 200 cases reported in the literature. Systemic involvement was carefully excluded using PET-CT and bone marrow biopsy, and diagnostic accuracy was supported by comprehensive immunohistochemical and molecular analyses. Limitations include the limited generalizability inherent to a single-case report and the lack of advanced MRI modalities, such as perfusion imaging or spectroscopy, which could have facilitated preoperative distinction from meningioma; cerebrospinal fluid analysis was not obtained, although there was no clinical or radiologic evidence of leptomeningeal involvement.

Alternative strategies, including biopsy followed by primary chemoradiotherapy, could have been considered; however, surgical resection provided rapid symptomatic relief, reduced mass effect, and enabled a definitive diagnosis, consistent with current practice for meningioma-mimicking dural lesions. The use of rituximab-based chemotherapy followed by focal radiotherapy was consistent with contemporary standards of care for high-grade PDL. This case is distinguished by its purely dural location, aggressive histology, radiologic mimicry of meningioma, and favorable outcome with multimodal therapy, reinforcing the evidence that PDL, even in high-grade forms, may achieve durable remission with appropriate treatment.

## Conclusions

Primary dural DLBCL is a rare and diagnostically challenging entity that frequently resembles meningioma on imaging, making histopathological confirmation essential. Multidisciplinary management, including surgical resection, rituximab-based chemotherapy, and radiotherapy, may be associated with durable disease control, as demonstrated in this case with 12 months of radiological remission. This report highlights the importance of maintaining clinical suspicion and employing a collaborative treatment approach to optimize outcomes in this uncommon but potentially treatable condition.
